# Supplementary material for: The mosaic of science: Disciplinary diversity and scientific prestige in research groups in Colombia
Source: PLoS One. 2026 Feb 25;21(2):e0343738. doi: 10.1371/journal.pone.0343738 (PMC12935232; doi:10.1371/journal.pone.0343738)
Supplement: S1 Table — (DOCX) [file pone.0343738.s001.docx]

# Supporting Information

S 1 Table. Median DIV by group rank and size, 2013-2021

|  | **2013** | **2014** | **2015** | **2017** | **2019** | **2021** |
| --- | --- | --- | --- | --- | --- | --- |
| *Agricultural sciences* | 0.0073 | 0.0080 | 0.0069 | 0.0071 | 0.0077 | 0.0077 |
| A | 0.0078 | 0.0108 | 0.0086 | 0.0072 | 0.0077 | 0.0080 |
| large |  | 0.0186 | 0.0125 | 0.0101 | 0.0109 | 0.0110 |
| medium | 0.0091 | 0.0065 | 0.0075 | 0.0061 | 0.0069 | 0.0066 |
| small | 0.0065 | 0.0074 | 0.0056 | 0.0055 | 0.0053 | 0.0064 |
| A1 | 0.0085 | 0.0076 | 0.0079 | 0.0081 | 0.0080 | 0.0086 |
| large | 0.0101 | 0.0121 | 0.0118 | 0.0118 | 0.0125 | 0.0110 |
| medium | 0.0092 | 0.0064 | 0.0059 | 0.0078 | 0.0073 | 0.0082 |
| small | 0.0062 | 0.0044 | 0.0060 | 0.0048 | 0.0042 | 0.0065 |
| B | 0.0080 | 0.0082 | 0.0064 | 0.0082 | 0.0108 | 0.0090 |
| large | 0.0120 | 0.0119 |  | 0.0117 | 0.0207 | 0.0152 |
| medium | 0.0063 | 0.0067 | 0.0072 | 0.0078 | 0.0066 | 0.0064 |
| small | 0.0057 | 0.0059 | 0.0056 | 0.0051 | 0.0052 | 0.0054 |
| C | 0.0063 | 0.0053 | 0.0053 | 0.0069 | 0.0073 | 0.0061 |
| large |  |  |  | 0.0071 | 0.0096 | 0.0085 |
| medium | 0.0081 | 0.0063 | 0.0063 | 0.0088 | 0.0063 | 0.0042 |
| small | 0.0044 | 0.0043 | 0.0042 | 0.0049 | 0.0059 | 0.0056 |
| Reconocido | 0.0058 | 0.0056 | 0.0053 | 0.0038 | 0.0035 | 0.0053 |
| large | 0.0085 |  |  |  |  |  |
| medium | 0.0039 |  | 0.0042 | 0.0028 | 0.0033 |  |
| small | 0.0050 | 0.0056 | 0.0064 | 0.0048 | 0.0037 | 0.0053 |
| *Engineering and technology* | *0.0066* | *0.0076* | *0.0082* | *0.0074* | *0.0070* | *0.0073* |
| A | 0.0057 | 0.0064 | 0.0079 | 0.0078 | 0.0077 | 0.0070 |
| large |  | 0.0058 | 0.0104 | 0.0108 | 0.0108 | 0.0106 |
| medium | 0.0070 | 0.0064 | 0.0087 | 0.0079 | 0.0073 | 0.0062 |
| small | 0.0044 | 0.0070 | 0.0047 | 0.0048 | 0.0049 | 0.0042 |
| A1 | 0.0064 | 0.0069 | 0.0068 | 0.0065 | 0.0066 | 0.0063 |
| large | 0.0062 | 0.0087 | 0.0079 | 0.0086 | 0.0092 | 0.0084 |
| medium | 0.0072 | 0.0062 | 0.0063 | 0.0062 | 0.0060 | 0.0059 |
| small | 0.0057 | 0.0060 | 0.0062 | 0.0046 | 0.0045 | 0.0047 |
| B | 0.0055 | 0.0088 | 0.0083 | 0.0083 | 0.0087 | 0.0073 |
| large | 0.0030 | 0.0125 | 0.0116 | 0.0106 | 0.0129 | 0.0106 |
| medium | 0.0072 | 0.0078 | 0.0074 | 0.0089 | 0.0080 | 0.0067 |
| small | 0.0063 | 0.0060 | 0.0058 | 0.0053 | 0.0051 | 0.0047 |
| C | 0.0096 | 0.0073 | 0.0103 | 0.0083 | 0.0066 | 0.0099 |
| large | 0.0130 |  | 0.0148 | 0.0117 | 0.0083 | 0.0184 |
| medium | 0.0085 | 0.0090 | 0.0102 | 0.0078 | 0.0058 | 0.0059 |
| small | 0.0073 | 0.0056 | 0.0058 | 0.0055 | 0.0056 | 0.0053 |
| Reconocido | 0.0050 | 0.0088 | 0.0074 | 0.0052 | 0.0051 | 0.0053 |
| medium | 0.0060 | 0.0091 |  | 0.0035 | 0.0061 | 0.0060 |
| small | 0.0041 | 0.0086 | 0.0074 | 0.0069 | 0.0040 | 0.0046 |
| *Humanities* | *0.0069* | *0.0070* | *0.0057* | *0.0056* | *0.0059* | *0.0060* |
| A | 0.0066 | 0.0071 | 0.0042 | 0.0052 | 0.0062 | 0.0065 |
| large | 0.0080 |  |  | 0.0059 | 0.0070 | 0.0087 |
| medium | 0.0071 | 0.0091 | 0.0044 | 0.0061 | 0.0061 | 0.0069 |
| small | 0.0048 | 0.0051 | 0.0041 | 0.0035 | 0.0057 | 0.0040 |
| A1 | 0.0089 | 0.0060 | 0.0062 | 0.0057 | 0.0060 | 0.0070 |
| large | 0.0097 |  | 0.0070 | 0.0078 | 0.0066 | 0.0085 |
| medium | 0.0107 | 0.0087 | 0.0057 | 0.0052 | 0.0062 | 0.0072 |
| small | 0.0063 | 0.0033 | 0.0058 | 0.0040 | 0.0053 | 0.0052 |
| B | 0.0065 | 0.0079 | 0.0056 | 0.0057 | 0.0061 | 0.0058 |
| large | 0.0094 | 0.0130 |  | 0.0046 | 0.0086 | 0.0071 |
| medium | 0.0061 | 0.0069 | 0.0069 | 0.0073 | 0.0057 | 0.0052 |
| small | 0.0041 | 0.0039 | 0.0044 | 0.0052 | 0.0041 | 0.0049 |
| C | 0.0048 | 0.0067 | 0.0060 | 0.0064 | 0.0061 | 0.0049 |
| large |  | 0.0123 |  |  | 0.0043 |  |
| medium | 0.0036 | 0.0034 | 0.0062 | 0.0072 | 0.0083 | 0.0058 |
| small | 0.0060 | 0.0045 | 0.0058 | 0.0057 | 0.0056 | 0.0039 |
| Reconocido | 0.0066 | 0.0074 | 0.0065 | 0.0054 | 0.0035 | 0.0056 |
| medium |  |  | 0.0088 | 0.0058 |  | 0.0052 |
| small | 0.0066 | 0.0074 | 0.0042 | 0.0050 | 0.0035 | 0.0059 |
| *Medical and health sciences* | *0.0085* | *0.0091* | *0.0083* | *0.0084* | *0.0080* | *0.0077* |
| A | 0.0115 | 0.0092 | 0.0077 | 0.0073 | 0.0086 | 0.0070 |
| large |  | 0.0138 | 0.0082 | 0.0081 | 0.0125 | 0.0079 |
| medium | 0.0156 | 0.0070 | 0.0078 | 0.0072 | 0.0072 | 0.0066 |
| small | 0.0075 | 0.0068 | 0.0070 | 0.0067 | 0.0061 | 0.0065 |
| A1 | 0.0091 | 0.0088 | 0.0082 | 0.0089 | 0.0090 | 0.0094 |
| large | 0.0112 | 0.0110 | 0.0099 | 0.0110 | 0.0117 | 0.0131 |
| medium | 0.0097 | 0.0076 | 0.0082 | 0.0094 | 0.0083 | 0.0082 |
| small | 0.0064 | 0.0077 | 0.0065 | 0.0063 | 0.0070 | 0.0070 |
| B | 0.0081 | 0.0080 | 0.0091 | 0.0093 | 0.0081 | 0.0086 |
| large | 0.0091 | 0.0103 | 0.0142 | 0.0155 | 0.0106 | 0.0122 |
| medium | 0.0081 | 0.0077 | 0.0072 | 0.0071 | 0.0079 | 0.0077 |
| small | 0.0071 | 0.0061 | 0.0059 | 0.0052 | 0.0057 | 0.0060 |
| C | 0.0068 | 0.0111 | 0.0110 | 0.0088 | 0.0072 | 0.0072 |
| large |  | 0.0175 | 0.0183 | 0.0132 | 0.0083 | 0.0095 |
| medium | 0.0063 | 0.0096 | 0.0086 | 0.0074 | 0.0075 | 0.0067 |
| small | 0.0073 | 0.0061 | 0.0061 | 0.0059 | 0.0057 | 0.0053 |
| Reconocido | 0.0069 | 0.0083 | 0.0055 | 0.0076 | 0.0066 | 0.0060 |
| large | 0.0086 |  | 0.0043 | 0.0111 |  | 0.0079 |
| medium |  | 0.0113 | 0.0063 | 0.0043 | 0.0093 | 0.0066 |
| small | 0.0052 | 0.0052 | 0.0058 | 0.0074 | 0.0039 | 0.0036 |
| *Natural sciences* | *0.0060* | *0.0075* | *0.0066* | *0.0077* | *0.0069* | *0.0074* |
| A | 0.0050 | 0.0072 | 0.0077 | 0.0068 | 0.0080 | 0.0079 |
| large | 0.0023 | 0.0101 | 0.0119 | 0.0087 | 0.0110 | 0.0127 |
| medium | 0.0072 | 0.0068 | 0.0069 | 0.0072 | 0.0079 | 0.0063 |
| small | 0.0054 | 0.0047 | 0.0043 | 0.0046 | 0.0050 | 0.0047 |
| A1 | 0.0061 | 0.0060 | 0.0067 | 0.0063 | 0.0063 | 0.0064 |
| large | 0.0081 | 0.0078 | 0.0091 | 0.0092 | 0.0085 | 0.0095 |
| medium | 0.0060 | 0.0062 | 0.0066 | 0.0058 | 0.0062 | 0.0063 |
| small | 0.0042 | 0.0040 | 0.0043 | 0.0040 | 0.0042 | 0.0033 |
| B | 0.0067 | 0.0066 | 0.0083 | 0.0085 | 0.0089 | 0.0082 |
| large | 0.0078 | 0.0061 | 0.0104 | 0.0125 | 0.0136 | 0.0123 |
| medium | 0.0066 | 0.0073 | 0.0090 | 0.0084 | 0.0075 | 0.0070 |
| small | 0.0059 | 0.0064 | 0.0054 | 0.0047 | 0.0057 | 0.0054 |
| C | 0.0062 | 0.0102 | 0.0050 | 0.0058 | 0.0055 | 0.0061 |
| large |  | 0.0171 | 0.0020 | 0.0029 |  |  |
| medium | 0.0062 | 0.0068 | 0.0070 | 0.0090 | 0.0057 | 0.0068 |
| small | 0.0062 | 0.0066 | 0.0060 | 0.0055 | 0.0053 | 0.0055 |
| Reconocido | 0.0061 | 0.0077 | 0.0046 | 0.0110 | 0.0055 | 0.0080 |
| large |  |  |  | 0.0198 | 0.0084 | 0.0145 |
| medium | 0.0064 | 0.0084 | 0.0046 | 0.0078 | 0.0040 | 0.0044 |
| small | 0.0058 | 0.0069 | 0.0045 | 0.0052 | 0.0041 | 0.0050 |
| *Social sciences* | *0.0056* | *0.0050* | *0.0053* | *0.0054* | *0.0061* | *0.0064* |
| A | 0.0062 | 0.0054 | 0.0059 | 0.0059 | 0.0059 | 0.0060 |
| large | 0.0071 | 0.0064 | 0.0076 | 0.0078 | 0.0077 | 0.0073 |
| medium | 0.0057 | 0.0046 | 0.0060 | 0.0057 | 0.0052 | 0.0059 |
| small | 0.0057 | 0.0054 | 0.0041 | 0.0043 | 0.0049 | 0.0048 |
| A1 | 0.0055 | 0.0048 | 0.0048 | 0.0046 | 0.0053 | 0.0052 |
| large | 0.0081 | 0.0055 | 0.0061 | 0.0059 | 0.0065 | 0.0061 |
| medium | 0.0037 | 0.0037 | 0.0046 | 0.0045 | 0.0054 | 0.0048 |
| small | 0.0047 | 0.0051 | 0.0038 | 0.0035 | 0.0040 | 0.0046 |
| B | 0.0051 | 0.0059 | 0.0051 | 0.0060 | 0.0054 | 0.0064 |
| large | 0.0055 | 0.0080 | 0.0043 | 0.0062 | 0.0054 | 0.0081 |
| medium | 0.0055 | 0.0054 | 0.0058 | 0.0068 | 0.0058 | 0.0064 |
| small | 0.0042 | 0.0042 | 0.0051 | 0.0049 | 0.0049 | 0.0048 |
| C | 0.0059 | 0.0048 | 0.0046 | 0.0070 | 0.0051 | 0.0071 |
| large | 0.0071 |  | 0.0031 | 0.0102 | 0.0045 | 0.0107 |
| medium | 0.0051 | 0.0053 | 0.0055 | 0.0054 | 0.0060 | 0.0058 |
| small | 0.0055 | 0.0043 | 0.0050 | 0.0054 | 0.0049 | 0.0047 |
| Reconocido | 0.0051 | 0.0034 | 0.0064 | 0.0036 | 0.0090 | 0.0073 |
| large |  |  |  | 0.0026 | 0.0143 | 0.0131 |
| medium | 0.0038 | 0.0029 | 0.0071 | 0.0038 | 0.0067 | 0.0042 |
| small | 0.0064 | 0.0040 | 0.0057 | 0.0044 | 0.0060 | 0.0047 |
