# Supplementary material for: The mosaic of science: Disciplinary diversity and scientific prestige in research groups in Colombia
Source: PLoS One. 2026 Feb 25;21(2):e0343738. doi: 10.1371/journal.pone.0343738 (PMC12935232; doi:10.1371/journal.pone.0343738)
Supplement: Attachment — Submitted filename: response to reviewers.docx [file pone.0343738.s003.docx]

Dear editor(s) and reviewers,

Thanks for your insights and recommendations to improve the manuscript. Please find below the reviewers’ questions, our response, and the corresponding addition to the manuscript.

Sincerely,

The authors.

# 1 Editor

## 1.1 Comment:

Please ensure that your manuscript meets PLOS ONE's style requirements, including those for file naming.

### 1.1.1 Response of the author(s):

Files were naming according to the guidelines.

## 1.2 Comment:

We notice that your supplementary table is included in the manuscript file. Please remove them and upload them with the file type 'Supporting Information'. Please ensure that each Supporting Information file has a legend listed in the manuscript after the references list.

### 1.2.1 Response of the author(s):

The Supporting Information section was excluded from the manuscript, leaving only the legend listed. It will be uploaded as a separate document.

## 1.3 Comment

If the reviewer comments include a recommendation to cite specific previously published works, please review and evaluate these publications to determine whether they are relevant and should be cited. There is no requirement to cite these works unless the editor has indicated otherwise.

### 1.3.1 Response of the author(s):

I added a novel study to the subject published this year. This study is quite aligned with the topic of the manuscript, coherently added to the literature review and discussed in the respective section.

## 1.4 Comment

Please review your reference list to ensure that it is complete and correct. If you have cited papers that have been retracted, please include the rationale for doing so in the manuscript text, or remove these references and replace them with relevant current references. Any changes to the reference list should be mentioned in the rebuttal letter that accompanies your revised manuscript. If you need to cite a retracted article, indicate the article’s retracted status in the References list and also include a citation and full reference for the retraction notice.

### 1.4.1 Response of the author(s):

I added a novel study to the subject published this year. This study is quite aligned with the topic of the manuscript, coherently added to the literature review and also discussed in the respective section.

# 2 Reviewer: 1

## 2.1 Comment:

I have reviewed your manuscript and found it to be well-written and presenting a compelling contribution to the field. The content is likely to be of significant interest to readers and researchers in this area.

I recommend ensuring that all references cited are current and up-to-date. This will help to contextualize your findings within the latest developments in the field.

### 2.1.1 Response of the author(s):

We sincerely appreciate the reviewer’s appreciation of the study.

Concerning the references, we added the following study published this year (2025) to the “Related literature” and “Discussion and conclusion” sections as follows:

*A comprehensive study indicates that academic diversity within scientific teams has increased, as demonstrated by metrics such as academic entropy, standard deviation, and disparity, particularly in STEM fields and high-income countries. Despite this, higher levels of academic diversity do not have a clear association with teams that produce disruptive innovations at higher rates [42].*

*In addition, as shown by Yang et al. [42], higher disciplinary diversity is associated with lower levels of scientific disruption since this diversity seems to be invested in consolidating existing knowledge rather than producing disruptive innovations.*

*[42] Yang AJ, Zhao SX, Deng S, Liu M, Bu Y, Ding Y. Quantifying the dynamics of research teams’ academic diversity. J Assoc Inf Sci Technol. 2025;76: 1719–1735. doi:10.1002/ASI.70023*

# 3 Reviewer: 2

## 3.1 Comment:

With abundant data and appropriate methods, the overall logic is clear. But the clarity of the charts in the article needs to be improved, for example, replacing Table 1 with a graphical form would be clearer.

In addition, regarding the unclear description of the dataset on Page 8, it is suggested to add a table describing the dataset and provide annual data statistics

### 3.1.1 Response of the author(s):

The Table aims to show a refined count of groups by category, fields, and year. Yet, to improve the clarity of the data, I added the following description and Figure:

*For visual aid and synthesis, we added Fig. 1 showing the percentage of groups by major disciplinary area and national rank per year.*

Fig. 1 Percentage of groups by major disciplinary area and national rank per year

*Also, I added this row to sum the total of groups by year (all fields):*

| Total (all disciplines) | 1,202 | 1,438 | 1,817 | 2,328 | 2,767 | 3,107 |
| --- | --- | --- | --- | --- | --- | --- |

# 4 Reviewer: 3

## 4.1 Comment

An identical manuscript is already published on Internet - Research Gate. (https://www.researchgate.net/publication/395028979_A_Little_Bit_of_This_A_Little_Bit_of_That_-_Disciplinary_Diversity_and_Scientific_Prestige_in_Research_Groups_of_Colombia). do now know it this is possible and it could be considered that ” Results reported have not been published elsewhere.” and important criteria of Plos publication.

### 4.1.1 Response of the author(s):

There is a preprint version of this manuscript in Zenodo, reposted in RG with Zenodo’s DOI: <https://doi.org/10.5281/zenodo.16995532>

In the submission system, the preprint DOI was reported to the editors. PLoS ONE preprint policy allows authors to publish their manuscript on preprint servers and also “*Preprint checks do not impact the decision regarding suitability for publication in PLOS One*”: <https://journals.plos.org/plosone/s/preprints#:~:text=Authors%20can%20choose%20to%20supply,or%20tables%20and%20their%20captions>.
