# Supplementary material for: The mosaic of science: Disciplinary diversity and scientific prestige in research groups in Colombia
Source: PLoS One. 2026 Feb 25;21(2):e0343738. doi: 10.1371/journal.pone.0343738 (PMC12935232; doi:10.1371/journal.pone.0343738)
Supplement: Attachment — Submitted filename: response to reviewers_2.docx [file pone.0343738.s004.docx]

# Second round

Dear editor(s) and reviewers,

Thanks for your insights and recommendations to improve the manuscript. Please find below the reviewers’ questions, our response, and the corresponding addition to the manuscript.

Sincerely,

The authors.

# 1 Reviewer #2

## 1.1 Comment

With abundant data and appropriate methods, the overall logic is clear. But the clarity of the charts in the article needs to be improved, for example, replacing Table 1 with a graphical form would be clearer.

In addition, regarding the unclear description of the dataset on Page 8, it is suggested to add a table describing the dataset and provide annual data statistics

### 1.1.1 Response of the author(s):

This comment was addressed in round 1. Find the response below:

The Table aims to show a refined count of groups by category, fields, and year. Yet, to improve the clarity of the data, I added the following description and Figure:

*For visual aid and synthesis, we added Fig. 1 showing the percentage of groups by major disciplinary area and national rank per year.*

Fig. 1 Percentage of groups by major disciplinary area and national rank per year

*Also, I added this row to sum the total of groups by year (all fields):*

| Total (all disciplines) | 1,202 | 1,438 | 1,817 | 2,328 | 2,767 | 3,107 |
| --- | --- | --- | --- | --- | --- | --- |

# 2 Reviewer # 4

## 2.1 Comment:

The discussion would benefit from a more explicit comparison between the effects of increasing group size and disciplinary diversity on scientific prestige. While both dimensions are analyzed, the manuscript could more clearly disentangle whether prestige gains are primarily driven by larger group size (for example through productivity, resource pooling, or visibility) rather than diversity per se, and how these two factors interact.

### 2.1.1 Response of the author(s):

We added this section to the Discussion along with the references:

*While group size and disciplinary diversity are concurrent attributes of successful research teams, their impact on scientific prestige is mediated by different processes. The positive correlation between group size and ranking is predominantly influenced by the attainment of ‘critical mass,’ wherein larger entities leverage the consolidation of social and financial resources, alongside expertise, to foster increased knowledge output and collaboration that is quantifiable by national evaluation standards [71–73]. In this context, the diversity of disciplines arises in part as a structural consequence of expansion, as larger groups encompass a broader spectrum of fields, rather than being the singular independent determinant of prestige. However, size significantly moderates the effect of diversity, therefore it indicates that while large groups possess the structural resilience to harness the benefits of cognitive heterogeneity, smaller groups may face ‘coordination penalties’ where the costs of integrating disparate disciplines outweigh the potential benefits, particularly in the absence of the resource buffers found in larger groups.*

*69.	Liu L, Yu J, Huang J, Xia F, Jia T. The dominance of big teams in China’s scientific output. Quantitative Science Studies. 2021;2: 350–362. doi:10.1162/qss_a_00099*

*70.	Zhu N, Liu C, Yang Z. Team Size, Research Variety, and Research Performance: Do Coauthors’ Coauthors Matter? J Informetr. 2021;15: 101205. doi:10.1016/j.joi.2021.101205*

*71.	Cook I, Grange S, Eyre-Walker A. Research groups: How big should they be? PeerJ. 2015;3: e989. doi:10.7717/peerj.989*

## 2.2 Comment:

The discussion remains closely tied to the variables analyzed and could be broadened by situating the findings within the wider literature on research performance. In particular, factors beyond disciplinary composition—such as leadership quality, coordination capacity, institutional support, access to funding, international collaboration, and evaluation incentives—may influence research group rankings and could act as mediators or confounding factors.

Relatedly, the manuscript could benefit from a more explicit consideration of “soft” factors, such as communication, conflict management, leadership, and collaborative norms. These elements may have an impact that is orthogonal to, larger than, or even confounding with disciplinary diversity and group size. Discussing how such team-level dynamics might condition the effectiveness of diversity would add depth to the analysis.

### 2.2.1 Response of the author(s):

This segment was added, also, with additional references:

*Harnessing the benefits of disciplinary diversity in science teams and research groups relies on factors here not explored, such as communication, conflict management, and leadership since the coordination costs associated with bridging disciplinary gaps are significant. Cases have found that successful cross-disciplinary groups overcome these coordination costs through a process of cultural and disciplinary assimilation [14]. Also, funding and institutional support act as key mediators that influence how these teams form and function. For instance, strong organizational models such as structured as consortia, provided the necessary scaffold for deep cross-disciplinary integration but also pressure to secure resources, therefore, promoting the “convergence shortcuts,” where teams opt for polymathic generalists rather than facing the coordination challenges of integrating distinct specialists, suggesting that the race to secure funding might incentivize expedient teaming strategies over the more difficult, yet potentially more transformative, work of deep cross-disciplinary coordination [13].*

*[13] Petersen AM, Ahmed ME, Pavlidis I. Grand challenges and emergent modes of convergence science. Humanit Soc Sci Commun. 2021;8: 194. doi:10.1057/s41599-021-00869-9*

*[14] Petersen AM, Majeti D, Kwon K, Ahmed ME, Pavlidis I. Cross-disciplinary evolution of the genomics revolution. Sci Adv. 2018;4. doi:10.1126/sciadv.aat4211*

## 2.3 Comment:

While the focus on Colombia is a clear strength of the paper, the discussion could more explicitly address the extent to which the results can be generalized to other South American countries or to national research systems more broadly.

### 2.3.1 Response of the author(s):

This segment was added, also, with additional references:

*While we rely in Colombian context and data, the structural constraints identified—such as the prevalence of incremental research agendas driven by funding insecurity and a historical emphasis on professional training over scientific inquiry—are not unique to Colombia, but also, other Latin American countries [63,72]. Like other emerging economies, the pressure to secure resources in a scarcity-driven environment incentivizes ‘conservative’ strategies where groups prioritize volume and consolidated topics over high-risk research, which more often requires disciplinary diversity [13,14]. Furthermore, the confirmation of the Matthew effect in our trajectory analysis suggests that the stratification of research groups is a systemic feature of metric-based national evaluations, applicable to other countries employing similar ranking mechanisms. Finally, the non-monotonic relationship between disciplinary diversity and scientific prestige underscores a universal ‘Team Science’ principle that requires further resources, strategies, and approaches to understand it in depth [44].*

*[72] UNESCO. National science, technology and innovation systems in Latin America and the Caribbean. Lemarchand GA, editor. Montevideo: UNESCO Office Montevideo; 2010. Available: https://unesdoc.unesco.org/ark:/48223/pf0000189823*

## 2.4 Comment:

- Abstract: Change “Leydesdorff et al.” to “Leydesdorff et al.’s”.

Edited.

- Abstract: Change “on an volatile” to “on a volatile”.

Edited.

- Introduction: In the sentence “Modern society and science face complex challenges,” please provide concrete examples of such challenges.

### 2.4.1 Response of the author(s):

This section was added:

*Collaboration among science teams is essential for addressing complex global challenges, such as climate change, pandemics, or extreme inequality.*

- Introduction: In the sentence beginning with “This collaboration emerges…”, do you refer to scientific collaborations in general? Please clarify.

### 2.4.2 Response of the author(s):

This section was added:

*Scientific collaboration emerges as a rich tapestry woven with a diverse thread of thought from individuals from different institutions, nations, and areas of expertise [1–3].*

- Introduction: Change “research in the field of the science of team science (SciTS) have examined” to “has examined”.

Edited.

- Related Literature (second-to-last paragraph): Please define the abbreviation STEM.

Edited.

- Methods 3.1 (Reference 44): The year in the R reference appears to correspond to the release date of the R version used. I would expect a newer version than 3.1 to have been used; please adjust accordingly.

*Using the citation function, I used the official citation provided by the system:*

*R Core Team (2021). R: A language and environment for statistical computing. R Foundation for Statistical Computing, Vienna, Austria. URL https://www.R-project.org/.*

- Section 3.2.2 (Sampling): The subtitle “Sampling” is misleading, as no actual sampling procedure is described. I suggest changing it to “Selection” or a similar term.

Edited to: Sample.

- Section 3.2.2, paragraph 3: The scientific ranks are introduced here, but their meaning and assessment are explained later in subsection 3.3.2. It would improve clarity to restructure these sections or to indicate explicitly that further details follow.

This section was added before Table 1:

*Also, in numeral 3.3.2, we will explain the ranks and their relationship with scientific prestige and performance.*

- Tables and Figures: All table and figure captions should end with a period.

Edited.

- Figure 2: The y-axis labels are very long. Consider abbreviating them, for example as “# RGs”.

Edited. Also, we made a more informative and condensed Figure.

- Equation (1): Please clarify what the subscript “d” refers to. If it indicates the group, it is unclear why N would also need a subscript.

### 2.4.3 Response of the author(s):

Edited as follows:

*For each research group* $i$*, we calculate the proportion of unique disciplines present relative to the total pool of disciplines identified across all groups. We formalized as follows (Equation 1):*

${RV}_{i}=\frac{n_{i}}{N}$ *(1)*

*Where* $n_{i}$ *represents the number of distinct disciplines within research groups* $i$ *and* $N$ *represents the total number of distinct disciplines appearing across the entire sample.*

## 2.5 Comment:

Equation (2): The meaning of the symbol for the average value is unclear and should be specified.

### 2.5.1 Response of the author(s):

Edited as follows:

*The second component, balance, is defined as the evenness of the distribution of researchers across the disciplines represented within a research group from a certain major disciplinary area. We measured this via* $Gini coefficient (G)$*. We formalize as follows (Equation 2):*

$G=\frac{\sum_{i=1}^{n} \sum_{j=1}^{n} |x_{i}-x_{j}|}{2n^{2}\bar{x}}$ *(2)*

*Where* $x_{i}$ *represents the number of researchers in discipline* $i$*, and* $n$ *is the total number of disciplines in a research group from a certain major disciplinary area, and* $\bar{x}$ *is the arithmetic mean of the number of researchers across all* $n$ *disciplines. If the* $G$ *of a group has equal representation across all disciplines it be lower (i.e., high balance), whereas if researchers are concentrated in a few disciplines (i.e., imbalance) the coefficient will be higher.*

## 2.6 Comment:

Equations (3, 4, 6, 7): The notation for scalar multiplication is inconsistent. Please use a single convention throughout (for example, a multiplication dot or a multiplication sign).

Edited. All notation was standardized as: “$\times$”

## 2.7 Comment:

Figure 3: Please explain the meaning of the different shapes used for Disparity. The color coding for Variety and Balance is intuitive, but the interpretation of Disparity is not clear.

### 2.7.1 Response of the author(s):

Edited as follows:

*Fig. 3 Cases of research groups with higher and lower diversity. Elaboration of the author based on Ref. [14]. Note: Variety is indicated by the number of distinct elements, each distinguished by specific color coding. Balance is represented by the proportional color composition; the dominance of a single hue denotes an uneven distribution or concentration of researchers within that discipline. Disparity is visualized through geometric shapes corresponding to major disciplinary areas—such as circles for Natural Sciences and triangles for Social Sciences—which illustrate the cognitive distance between the disciplines.*

## 2.8 Comment:

Table 4: This table occupies a substantial amount of space, but its content could likely be summarized in a few sentences. Please consider removing it.

### 2.8.1 Response of the author(s):

We consider that Table 4 effectively illustrates two contrasting extremes of diversity through specific groups examples. We believe this information is valuable for readers engaging with the text, as it provides concrete context for the study's theoretical concepts.

## 2.9 Comment

Figures 4, 5, and 6: These figures show the development over time of median DIV and related measures for different scientific areas and group sizes. However, the time axis is not evenly spaced, which visually overstates some effects. The time axis should be adjusted. In addition, there is a large amount of unused vertical space; adjusting the y-axis limits would improve readability.

Figure 5: Since there is only one median number of researchers per group rank, the legends could be combined. As currently shown, the legend suggests additional combinations that are not present. If only a subset is displayed, this should be clearly stated in the caption.

### 2.9.1 Response of the author(s):

This feedback allowed us to synthesize Figures 4, 5, and 6 into one cohesive figure, aiming to represent their essential idea:

*Fig. 4 illustrates the temporal evolution of the median DIV of research groups across areas and size types for the period between 2013 and 2021. The S 1 Table reports in more detail the median DIV by area, group rank, and size. For the sake of interpretability, we exclude the D and ‘Recognized’ ranks (~5% of the total sample).*

*Diversity trends varied significantly across research areas between 2013 and 2021. In most fields—specifically Medical and Health Sciences, Engineering and Technology, Natural Sciences, and Humanities—the median diversity index (DIV) in 2021 decreased compared to 2013 levels. Conversely, Agricultural Sciences and Social Sciences exhibited a marginal increase in median DIV over the same period. When analyzing trends by group classification, higher-ranked groups (A1 and A), which are characterized by a larger median number of researchers, have recently shown an upward trajectory in diversity, whereas groups in categories B and C have generally declined.*

*Despite these temporal trends, the relationship between a research group's disciplinary diversity and its national rank is non-linear and moderated by factors such as scientific area and group size. While higher-ranked groups (A1 and A) display increasing trajectories, the highest overall median disciplinary diversity is observed in B-ranked groups, followed by categories A and C. Consequently, the aggregated data do not support a simple direct correlation between greater disciplinary diversity and higher scientific prestige. Instead, field-specific patterns emerge: a positive association between diversity and prestige is prominent in the Humanities and Medical and Health Sciences. In contrast, an inverse relationship characterizes the Social Sciences, Natural Sciences, Agricultural Sciences, and Engineering and Technology, where groups exhibiting higher diversity are more frequently found in lower national ranks.*

## 2.10 Comment:

Figure 10: Since median DIV values are used for the subsequent statistical tests, it would be helpful to also visualize the corresponding variability (for example, standard deviations or interquartile ranges).

### 2.10.1 Response of the author(s):

Added:

*The S 1 Table, reports in more detail the median DIV by area, group rank, and size.*

# 3 Reviewer # 5

## 3.1 Comment:

- I found it difficult to identify the definition of rank (A, A1, B, C) – which actually occurs in Section 3.3.2, well after they are mentioned in 3.2.2. These should be made more explicitly clear up front, so that one does not need to infer that A1 > A > B > C; and also, to address why is there an A1 but no B2, etc;

### 3.1.1 Response of the author(s):

We added the following text in the section 3.1 Methods, more explicitly and up front:

*We use the national classification system, which evaluates research groups across five hierarchical tiers—A1, A, B, C, and “Recognized”, being A1 groups those of the highest prestige—based on criteria defined by MinCiencias, which include research output, collaboration, and contributions to researcher training and innovation. This variable is explained in detail in numeral the numeral 3.3.2.*

Also, we added the following text in numeral 3.3.2:

*In essence, the rank is a hierarchical classification where the "A1" category represents an elite tier distinguished by a rigorous double filter, requiring concurrent placement in the top quartile (Q1) for both Group and Top Product indicators. In contrast, categories "A" and "B" occupy broader competitive ranges—encompassing the top 50% and 75% respectively—and mandate only the presence of high-quality products rather than specific quartile positioning for those outputs. Finally, Category "C" operates distinctively as a non-competitive baseline based on a checklist of minimum requirements, such as existence duration and human resource formation; this design positions it as a transitional entry point intended to foster advancement toward consolidation, thereby negating the need for internal subdivisions like C1 or C2, as well as in the B rank.*

## 3.2 Comment:

Also the first several Table/Plots would suggest that A>A1, which could be confusing.

### 3.2.1 Response of the author(s):

All plots and tables were updated as suggested, placing A1 > A > B > C > D > “Recognized”.

## 3.3 Comment:

- I’m not sure what is the objective or value of the flippant title – I would recommend to remove the header “A Little Bit of This, A Little Bit of That”, as it is vague and does not contribute anything substantial or informative.

### 3.3.1 Response of the author(s):

After noting the author's valuable advice on reducing informal language and preserving the research topic's engaging and general nature in the title, it was edited as follows:

*The Mosaic of Science — Disciplinary Diversity and Scientific Prestige in Research Groups of Colombia*

## 3.4 Comment

- Regarding the single-sentence statement - “In sum, evidence showed that, while diversity may offer benefits in specific, cognitively demanding contexts, it is not a universal performance enhancer” – here are two studies providing strong evidence in support of a causal link between team disciplinary diversity and broader citation impact:

Petersen AM, Majeti D, Kwon K, Ahmed ME, Pavlidis I. Cross-disciplinary evolution of the genomics revolution. Science advances. 2018 Aug 15;4(8):eaat4211.

Petersen AM, Ahmed ME, Pavlidis I. Grand challenges and emergent modes of convergence science. Humanities and Social Sciences Communications. 2021 Aug 4;8(1):1-5.

### 3.4.1 Response of the author(s):

Both studies were cited and also discussed in the discussion:

*Harnessing the benefits of disciplinary diversity in science teams and research groups relies on factors here not explored, such as communication, conflict management, and leadership since the coordination costs associated with bridging disciplinary gaps are significant. Cases have found that successful cross-disciplinary groups overcome these coordination costs through a process of cultural and disciplinary assimilation [14]. Also, funding and institutional support act as key mediators that influence how these teams form and function. For instance, strong organizational models such as structured as consortia, provided the necessary scaffold for deep cross-disciplinary integration but also pressure to secure resources, therefore, promoting the “convergence shortcuts,” where teams opt for polymathic generalists rather than facing the coordination challenges of integrating distinct specialists, suggesting that the race to secure funding might incentivize expedient teaming strategies over the more difficult, yet potentially more transformative, work of deep cross-disciplinary coordination [13].*

*[13] Petersen AM, Ahmed ME, Pavlidis I. Grand challenges and emergent modes of convergence science. Humanit Soc Sci Commun. 2021;8: 194. doi:10.1057/s41599-021-00869-9*

*[14] Petersen AM, Majeti D, Kwon K, Ahmed ME, Pavlidis I. Cross-disciplinary evolution of the genomics revolution. Sci Adv. 2018;4. doi:10.1126/sciadv.aat4211*

**§**

# First round

Dear editor(s) and reviewers,

Thanks for your insights and recommendations to improve the manuscript. Please find below the reviewers’ questions, our response, and the corresponding addition to the manuscript.

Sincerely,

The authors.

# 4 Editor

## 4.1 Comment:

Please ensure that your manuscript meets PLOS ONE's style requirements, including those for file naming.

### 4.1.1 Response of the author(s):

Files were naming according to the guidelines.

## 4.2 Comment:

We notice that your supplementary table is included in the manuscript file. Please remove them and upload them with the file type 'Supporting Information'. Please ensure that each Supporting Information file has a legend listed in the manuscript after the references list.

### 4.2.1 Response of the author(s):

The Supporting Information section was excluded from the manuscript, leaving only the legend listed. It will be uploaded as a separate document.

## 4.3 Comment

If the reviewer comments include a recommendation to cite specific previously published works, please review and evaluate these publications to determine whether they are relevant and should be cited. There is no requirement to cite these works unless the editor has indicated otherwise.

### 4.3.1 Response of the author(s):

I added a novel study to the subject published this year. This study is quite aligned with the topic of the manuscript, coherently added to the literature review and discussed in the respective section.

## 4.4 Comment

Please review your reference list to ensure that it is complete and correct. If you have cited papers that have been retracted, please include the rationale for doing so in the manuscript text, or remove these references and replace them with relevant current references. Any changes to the reference list should be mentioned in the rebuttal letter that accompanies your revised manuscript. If you need to cite a retracted article, indicate the article’s retracted status in the References list and also include a citation and full reference for the retraction notice.

### 4.4.1 Response of the author(s):

I added a novel study to the subject published this year. This study is quite aligned with the topic of the manuscript, coherently added to the literature review and also discussed in the respective section.

# 5 Reviewer: 1

## 5.1 Comment:

I have reviewed your manuscript and found it to be well-written and presenting a compelling contribution to the field. The content is likely to be of significant interest to readers and researchers in this area.

I recommend ensuring that all references cited are current and up-to-date. This will help to contextualize your findings within the latest developments in the field.

### 5.1.1 Response of the author(s):

We sincerely appreciate the reviewer’s appreciation of the study.

Concerning the references, we added the following study published this year (2025) to the “Related literature” and “Discussion and conclusion” sections as follows:

*A comprehensive study indicates that academic diversity within scientific teams has increased, as demonstrated by metrics such as academic entropy, standard deviation, and disparity, particularly in STEM fields and high-income countries. Despite this, higher levels of academic diversity do not have a clear association with teams that produce disruptive innovations at higher rates [42].*

*In addition, as shown by Yang et al. [42], higher disciplinary diversity is associated with lower levels of scientific disruption since this diversity seems to be invested in consolidating existing knowledge rather than producing disruptive innovations.*

*[42] Yang AJ, Zhao SX, Deng S, Liu M, Bu Y, Ding Y. Quantifying the dynamics of research teams’ academic diversity. J Assoc Inf Sci Technol. 2025;76: 1719–1735. doi:10.1002/ASI.70023*

# 6 Reviewer: 2

## 6.1 Comment:

With abundant data and appropriate methods, the overall logic is clear. But the clarity of the charts in the article needs to be improved, for example, replacing Table 1 with a graphical form would be clearer.

In addition, regarding the unclear description of the dataset on Page 8, it is suggested to add a table describing the dataset and provide annual data statistics

### 6.1.1 Response of the author(s):

The Table aims to show a refined count of groups by category, fields, and year. Yet, to improve the clarity of the data, I added the following description and Figure:

*For visual aid and synthesis, we added Fig. 1 showing the percentage of groups by major disciplinary area and national rank per year.*

Fig. 1 Percentage of groups by major disciplinary area and national rank per year

*Also, I added this row to sum the total of groups by year (all fields):*

| Total (all disciplines) | 1,202 | 1,438 | 1,817 | 2,328 | 2,767 | 3,107 |
| --- | --- | --- | --- | --- | --- | --- |

# 7 Reviewer: 3

## 7.1 Comment

An identical manuscript is already published on Internet - Research Gate. (https://www.researchgate.net/publication/395028979_A_Little_Bit_of_This_A_Little_Bit_of_That_-_Disciplinary_Diversity_and_Scientific_Prestige_in_Research_Groups_of_Colombia). do now know it this is possible and it could be considered that ” Results reported have not been published elsewhere.” and important criteria of Plos publication.

### 7.1.1 Response of the author(s):

There is a preprint version of this manuscript in Zenodo, reposted in RG with Zenodo’s DOI: <https://doi.org/10.5281/zenodo.16995532>

In the submission system, the preprint DOI was reported to the editors. PLoS ONE preprint policy allows authors to publish their manuscript on preprint servers and also “*Preprint checks do not impact the decision regarding suitability for publication in PLOS One*”: <https://journals.plos.org/plosone/s/preprints#:~:text=Authors%20can%20choose%20to%20supply,or%20tables%20and%20their%20captions>.
